# Influence of Physical-Chemical Soil Parameters on Microbiota Composition and Diversity in a Deep Hyperarid Core of the Atacama Desert

**DOI:** 10.3389/fmicb.2021.794743

**Published:** 2022-02-07

**Authors:** Bárbara Fuentes, Alessandra Choque, Francisco Gómez, Jaime Alarcón, Eduardo Castro-Nallar, Franko Arenas, Daniel Contreras, Ramona Mörchen, Wulf Amelung, Claudia Knief, Ghazal Moradi, Erwin Klumpp, Claudia P. Saavedra, Jörg Prietzel, Wantana Klysubun, Francisco Remonsellez, Roland Bol

**Affiliations:** ^1^Departamento de Ingeniería Química, Universidad Católica del Norte, Antofagasta, Chile; ^2^Programa de Doctorado en Ciencias Mención Geología, Universidad Católica del Norte, Antofagasta, Chile; ^3^Center for Bioinformatics and Integrative Biology, Universidad Andres Bello, Santiago, Chile; ^4^Institute of Crop Science and Resource Conservation, Soil Science and Soil Ecology, University of Bonn, Bonn, Germany; ^5^Institute of Crop Science and Resource Conservation, Molecular Biology of the Rhizosphere, University of Bonn, Bonn, Germany; ^6^Institute of Bio and Geosciences, Agrosphere (IBG-3), Forschungszentrum Jülich GmbH, Jülich, Germany; ^7^Laboratorio de Microbiología Molecular, Departamento de Ciencias Biológicas, Facultad de Ciencias de la Vida, Universidad Andrés Bello, Santiago, Chile; ^8^Wissenschaftszentum Weihenstephan, Technical University München, Freising, Germany; ^9^Synchrotron Light Research Institute, Nakhon Ratchasima, Thailand; ^10^Centro de Investigación Tecnológica del Agua en el Desierto-CEITSAZA, Universidad Católica del Norte, Antofagasta, Chile; ^11^School of Natural Sciences, Environment Centre Wales, Bangor University, Bangor, United Kingdom

**Keywords:** deep soil, physicochemical properties, microbiota, hyperarid soil, Atacama Desert

## Abstract

The extreme environmental conditions and lack of water on the soil surface in hyperarid deserts hamper microbial life, allowing only highly specialized microbial communities to the establish colonies and survive. Until now, the microbial communities that inhabit or have inhabited soils of hyperarid environments at greater depths have been poorly studied. We analyzed for the first time the variation in microbial communities down to a depth of 3.4 m in one of the driest places of the world, the hyperarid Yungay region in the Atacama Desert, and we related it to changes in soil physico-chemical characteristics. We found that the moisture content changed from 2 to 11% with depth and enabled the differentiation of three depth intervals: (i) surface zone A (0–60 cm), (ii) intermediate zone B (60–220 cm), and (iii) deep zone C (220–340 cm). Each zone showed further specific physicochemical and mineralogical features. Likewise, some bacterial phyla were unique in each zone, i.e., members of the taxa *Deinococcota*, *Halobacterota*, and *Latescibacterota* in zone A; *Crenarchaeota*, *Fusobacteriota*, and *Deltaproteobacterium* Sva0485 in zone B; and *Fervidibacteria* and *Campilobacterota* in zone C, which indicates taxon-specific preferences in deep soil habitats. Differences in the microbiota between the zones were rather abrupt, which is concomitant with abrupt changes in the physical-chemical parameters. Overall, moisture content, total carbon (TC), pH, and electric conductivity (EC) were most predictive of microbial richness and diversity, while total sulfur (TS) and total phosphorous (TP) contents were additionally predictive of community composition. We also found statistically significant associations between taxa and soil properties, most of which involved moisture and TC contents. Our findings show that under-explored habitats for microbial survival and existence may prevail at greater soil depths near water or within water-bearing layers, a valuable substantiation also for the ongoing search for biosignatures on other planets, such as Mars.

## Introduction

The hyperarid region of the Atacama Desert is in the south of this desert as part of the Aguas Blancas (AB) basin. This region is surrounded by mountain ranges that limit its extension to the Coastal Cordillera to the west and the Cordillera de Domeyko to the east (Bonilla, 1972). The mean annual precipitation is less than 1 mm yr^–1^ (Houston and Hartley, 2003; Houston, 2006), with precipitation (Pp) to potential evapotranspiration (PET) ratios of Pp/PET < 0.05 (UNEP, 1997). While the crest line of the coastal range blocks any incoming marine fog by approximately 100 km, this promotes a “fog shadow” in the area where some “Mars-like soils” mostly dominate (Navarro-González et al., 2003; Gómez-Silva et al., 2008). At present, this hyperarid core of the desert is also devoid of plant growth. However, several studies have shown that the Atacama Desert had humid periods in the past that varied from the current arid or hyperarid conditions (Sáez et al., 2016; Pfeiffer et al., 2018; Ritter et al., 2018).

Despite the hyperarid conditions of the soils, the existence of past and recent signals of life has been shown. A study by Wang et al. (2021) identified fingerprints of past biological activity in the Atacama Desert using phosphate oxygen (^18^O) isotopes. Others works have reported the existence of unexpectedly large microbial populations in the hyperarid soils, especially in very particular and extreme habitats, such as the underside of quartz rocks (Warren-Rhodes et al., 2006), inside of halite evaporates (Wierzchos et al., 2006), fumaroles at the Andes Mountains, and in caves of the Coastal range (Azua-Bustos et al., 2012; Bull and Asenjo, 2013). Liquid water availability and solar radiation are the main life-controlling factors in the Atacama Desert (Bull and Asenjo, 2013). Despite the significantly challenging environmental conditions, microorganisms and organic matter have also been detected in the surface and subsurface layers of the hyperarid soils of the Atacama Desert (Mörchen et al., 2019; Warren-Rhodes et al., 2019; Knief et al., 2020). In particular, the surface soils of the Yungay area have been widely studied regarding their microbial diversity. It has been shown that microbial life can even be detected under low water availability (Connon et al., 2007; Azua-Bustos et al., 2012, 2015, 2018, 2020; Fletcher et al., 2012; Crits-Christoph et al., 2013; Neilson et al., 2017; Warren-Rhodes et al., 2019; Schulze-Makuch et al., 2021). Furthermore, DNA-based sequence analyses revealed that bacterial communities in this hyperarid core display varied but relatively low levels of diversity and that water availability and salt contents are key factors shaping the hyperarid Atacama soil microbiome (Crits-Christoph et al., 2013; Warren-Rhodes et al., 2019; Knief et al., 2020). While Neilson et al. (2017) found that community richness and diversity were positively correlated with soil humidity, other studies highlighted that sudden ‘large’ inputs of water into regions that had remained hyperarid for millions of years can be harmful to surface soil microbial species, especially those specifically adapted to extremely low water availability (Azua-Bustos et al., 2018). Although biosignatures and microbial life have been poorly studied in the depth of this hyperarid environment, the first evidence for metabolically active microbial communities and patterns specifically adapted to this harsh climate exists (Schulze-Makuch et al., 2018). In this matter, Warren-Rhodes et al. (2019) analyzed the spatial distribution of microbial communities between 0 and 80 cm; surprisingly, they found significant subsurface microbial communities that existed related to residual sediment moisture. This study also highlighted the influence of soluble salts and mineralogy on water availability and likely microbial life in the deeper parts of the soil. More recently, Azua-Bustos et al. (2020) found a diverse microbial community and biosignatures in humid smectite-rich subsurface layers in the hyperarid core of the Atacama Desert (at 30-40 cm depth), and Schulze-Makuch et al. (2021) showed that hypolithically colonized rocks are microbial hotspots in this desert environment.

Considering the varying minerals detected in various soil layers of the hyperarid core, such as halite, calcite, smectite, montmorillonite, dolomite, and others (Ewing et al., 2006; Fuentes et al., 2014; Azua-Bustos et al., 2020), it can be expected that the overall physicochemical properties and the microbial communities would also differ along these various layers within a soil profile. Nevertheless, microbial life in the subsurface soil of hyperarid Yungay and its relationship to physicochemical variations in soil properties have been poorly studied thus far. Therefore, we aimed to dig deeper into hyperarid soil, unraveling the deeper soil microbiota and relating it to variations in soil properties.

## Materials and Methods

### Study Site in the Hyperarid Core of the Atacama Desert

**FIGURE 1:**
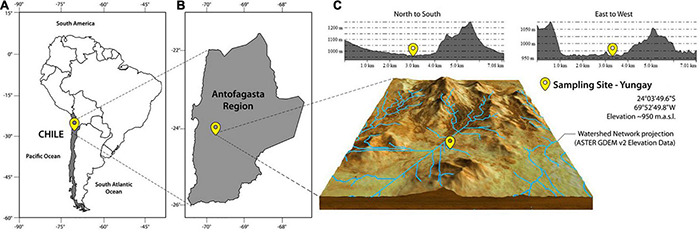
Location of the study site in Yungay, in the hyperarid core, Atacama Desert. **(A)** Chile, **(B)** Antofagasta Region, **(C)** Yungay in the hyperarid core.

**FIGURE 2:**
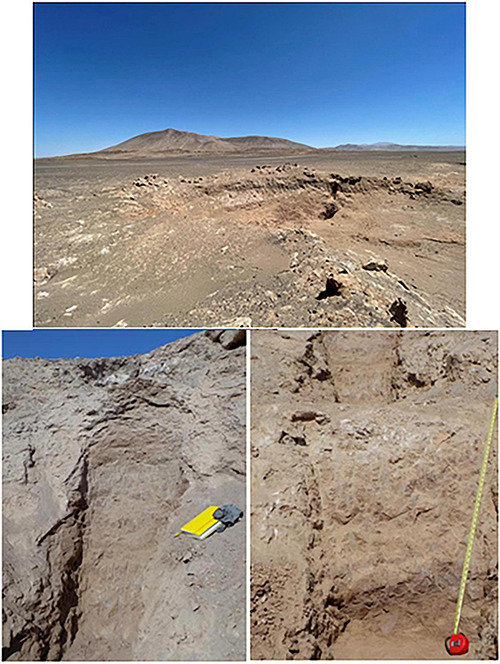
Images of soil sampling site in Yungay hyperarid core, Atacama Desert.

The study site is in the Yungay area of the Aguas Blancas (AB) basin (between 24°01′ and 24°16′ S; elevation range of 1,000–1,300 m.a.s.l.). It is situated southeast of Antofagasta within the hyperarid core of the Atacama Desert. The positional coordinates are 24°03′49.6″S, 69°52′49.8″W (Figure 1). Note that the surrounding area has also been studied by Pfeiffer et al. (2018) and Azua-Bustos et al. (2020). Some of our findings are in accordance with Pfeiffer et al. (2018), e.g., anhydrite polygons at the surface and needle fiber calcite in the soil profile. A soil scarp generated previously by a trench excavator was used in this study. Within the escarpment, we dug into the surface to freshly expose the upper part (2.2 m) of profile. Subsequently, we then continue digging below the surface till a depth of 1.2 m. Thus obtaining an overall profile of 3.4 m (Figure 2).

### Physical-Chemical and Mineralogical Characterization of the Soil Profile

Soil samples were taken in March 2017. The soil profile was sampled at 10 cm depth intervals until a final depth of 340 cm. Before the sampling procedure, the surface area was cleaned and cut back into its exposure. We took soil samples immediately after excavating a specific depth layer to avoid effects of the exposure of the soil to ambient air and temperature. Soil samples were manually taken and stored in plastic bags. Additionally, soil samples for microbiological analyses were collected in sterile tubes by using plastic gloves and shovels properly cleaned with ethanol to avoid contamination. All samples were taken to the laboratory in Antofagasta and Germany to conducted physicochemical, mineralogical, and microbiological analyses. The moisture content (MC) of the soil samples was measured immediately in the laboratory in Antofagasta by drying soil at 105°C in an oven and calculating the loss of weight after drying. Electric conductivity (EC) and pH were measured in a soil-distilled water suspension with a solution ratio of 1:2.5. The total contents of C, N, S, and TOC were determined by elemental analysis employing approx. 20 mg of sample material (Vario Micro Cube, Elementar, Hanau, Germany; ISO 10694, 1995). For the determination of TOC contents, inorganic carbon was removed with 20% HCl for 2 h. Total P, reactive P that corresponds to inorganic P, and unreactive P (URP) (which includes organic P and polyphosphates in soils) were determined by the extraction method proposed by Saunders and Williams (1955). The P concentration was measured by the method of Murphy and Riley (1962). P in the soil samples was also analyzed to determine the contribution of different P forms to total P by K-edge XANES spectroscopy at beamline 8 of the Synchrotron Light Research Institute (SLRI) in Nakhon Ratchasima, Thailand (Klysubun et al., 2019). XANES spectra acquisitions and the evaluation of the standard compounds were performed according to Werner and Prietzel (2015). The reference compounds were published by Prietzel et al. (2016). Sequential P fractionation was conducted according to Hedley et al. (1982) with modifications. Briefly, 0.3 g of soil samples was shaken in 30 ml of extractant solution. The order of extractants used was deionized water, 0.5 M NaHCO_3_ (pH 8.5), 0.1 M NaOH, and 1 M HCl. P not extracted by these solutions was classified as residual P. The amount of inorganic P (Pi) in each extract was determined by Murphy and Riley (1962). Selected soil samples of the soil profile were subjected to X-ray diffraction (XRD) to analyze its mineral compounds. XRD analysis was conducted with a Siemens Model D5000 diffractometer (Cu Kα1).

### Statistical Analysis of Soil Physicochemical Data

Based on the MC distribution along the soil profile, it was possible to distinguish and propose three distinct zones in the soil profile: surface zone A (0–60 cm), intermediate zone B (60–220 cm), and deep zone C (220–340 cm). For each zone, a linear regression was calculated for moisture content depending on depth. All data obtained were tested for normality of distribution by using a Shapiro–Wilk test (*n* < 50 for each defined zone). As the distribution was not normal, a non-parametric Friedman test for related samples was used to determine significant variations (*p* < 0.05) in the distribution of MC, TC, TOC, TN, TS and TP between the proposal zonification. All statistical analyses were performed with IBM SPSS Statistical software version 21.

### DNA Extraction, Sequencing and Taxonomic Analysis

Twenty grams of soil was suspended in 40 ml nuclease-free water in sterile Falcon tubes as described previously (Finstad et al., 2017) and then filtered through a 0.22 μm sterilized filter (Millipore). DNA was extracted from the material on filter paper using the QIAGEN DNeasy PowerSoil Kit according to the manufacturer’s instructions. The quantity of DNA extracted was measured using a Qubit fluorimeter with high sensitivity reagent kit. The 16S rRNA gene was amplified using primers 515F and 806R, targeting the V4 region of the 16S rRNA gene with approx. 235 bp amplicon length (Caporaso et al., 2012). Sequencing was performed on an Illumina MiSeq platform in a 150 bp paired-end cycle run. Sequencing was performed at the Environmental Sample Preparation and Sequencing Facility, Argonne National Laboratory. Samples were demultiplexed using the split_libraries_fastq.py module from QIIME 1.9 [16S: –barcode_type 12; ITS1: –rev_comp_barcode] (Caporaso et al., 2010), and amplicon sequence variants (ASVs) were inferred with DADA2 v1.10.1 (Callahan et al., 2016) using [truncLen = c(140,135), maxN = 0, maxEE = c(2,2), truncQ = 2, rm.phix = TRUE]. Error rate learning, dereplication, and merging were performed using default settings. After building an ASV table and removing chimeras, taxonomic assignment was obtained by analyzing reads against the Silva v138 database (Quast et al., 2013) using DADA2 Ribosomal Database Project’s (RDP) naive Bayesian classifier (Wang et al., 2007). ASVs identified as Eukarya, Chloroplast, Mitochondria and *Escherichia/Shigella*, *Staphylococcus*, *Corynebacterium*, *Lactobacillus* and *Streptococcus* were removed (Eisenhofer et al., 2019). Moreover, the R package decontam was used to identify and remove contaminants using the “frequency” and “prevalence” methods (Davis et al., 2018). Finally, samples with fewer than 1000 reads were discarded to avoid relying on taxonomic classification with low support. Water blanks and mock communities (Zymo microbiomics standard) were included to assess contamination and accuracy. ASV sequences were aligned with DECIPHER v3.8 (Wright, 2016).

### Statistical Analysis of Amplicon Data

All statistical analyses were conducted in R v3.5.2 and RStudio v1.1.463 (RStudio Team, 2016) using ampvis2 v2.4.5 (Andersen et al., 2018), microbiomeSeq v0.1,^1^ phyloseq v1.26.1 (McMurdie and Holmes, 2013), stats v3.5.2 (R Core Team, 2018), effects v4.1-4 (Fox, 2003) and bipartite v2.14 (Dormann et al., 2008). Plots were generated using ggplot2 v3.1.1 (Wickham, 2016) and basic R functions. The alpha diversity indices Chao1, Shannon and Fisher were calculated using phyloseq and subjected to normality tests (Shapiro–Wilk). Generalized linear models (GLMs) were built to test the effects of soil variables on alpha diversity indices. This was done using the “glm” function for the stats package, with the argument family = “Gaussian” and a p value threshold of <0.05 for significant predictors. All models were plotted using the R package effects, and residuals were analyzed for normality and homoscedasticity. Ampvis2 was used to perform a redundancy analysis (RDA) on Hellinger-transformed ASV abundances constrained by the soil zone. The argument envfit_numeric was added to display numerical soil variables as vectors. The MicrobiomeSeq package was used to evaluate the relationship between the taxa agglomerated to their best taxonomy and numerical soil variables based on Pearson’s correlation, adjusting for multiple testing using the Benjamini-Hochberg correction (0.05 p value threshold) and for taxa + groups. Finally, for a two-dimensional matrix between taxa and sample origin (Zone), a bipartite network analysis was performed to visualize the distribution of phyla among soil zones based on their relative abundances using the plot web function from the bipartite R package (Dormann et al., 2008) with the default method cca, which leads to as few crossings of interactions as possible.

## Results

### Characterization of the Soil Profile

**FIGURE 3:**
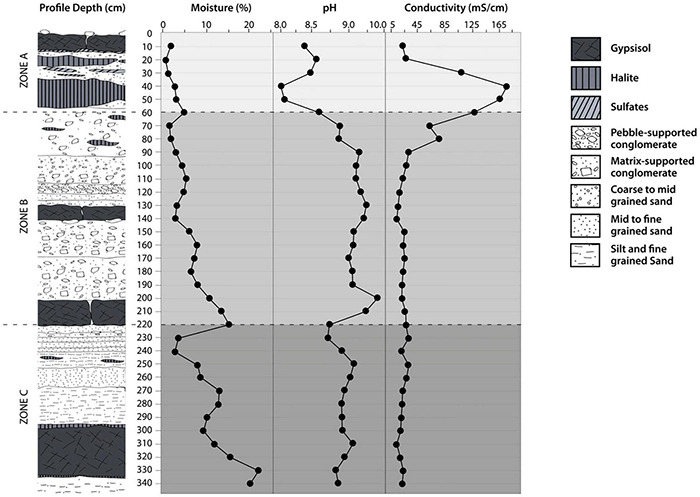
Distribution of the soil profile parameters at the Yungay site in the Atacama Desert. Moisture content (%), pH value and electric conductivity (EC).

According to the World Reference Base for Soil Resources, the soil profile can be classified as a Gypsisol. The profile and the complete description are given in Supplementary Figure 1. At the surface of the profile, fine sediments with desiccation cracks were recognized to develop over a massive chloride and sulfate salt layer with a thickness of 0.5 m. The sediments were finer below 2 m depth, and the soil particles had higher moisture contents than in the upper layers. The soil MC increased along with the soil profile (Figure 3). Considering that liquid water availability is one of the main life-controlling factors in the Atacama Desert (Bull and Asenjo, 2013), we propose three major zones in the profile based on linear regression analysis of the MC indicating changes with depth (Figure 3). These three proposed zones were surface zone A (0–60 cm, n: 6) with a slope of 0.066% moisture cm^–1^ R2: 0.71; intermediate zone B (60–220 cm, n: 16) with a slope of 0.073% moisture cm^–1^ R2: 0.80; and deep zone C (220–340 cm, n: 12) with a slope of 0.143% moisture cm^–1^ R2: 0.77. Moreover, soil MC increased significantly (*p* < 0.05, Friedman test) in the three proposal zones. In surface zone A, the MC increased from 0.66 to 5%. Additionally, we identified an evaporation layer between 0 and 20 cm within zone A, reflected by the lowest moisture content values registered in the soil profile (0.66%). In intermediate zone B, the moisture content increased to 15% at the 220 cm soil depth. In deep zone C, the moisture content reached a maximum of 21% at 330 cm soil depth. A local minimum of 3% moisture content between 220 and 240 cm depth was noted.

Within the three zones, we found different minerals (Supplementary Table 1). In surface zone A, the predominant minerals were halite, bassanite, anhydrite, albite, and calcite albite. Intermediate zone B was dominated by anorthite, calcite, albite, montmorillonite, and orthoclase. Deep zone C was composed of a wet clay-rich soil layer with high amounts of muscovite (approximately 20%) and lower abundances of chlorite (approximately 4%) (Supplementary Table 1). The values of pH along the soil profile were alkaline. However, it was also possible to detect significant differences in the three-zone intervals (*p* < 0.05, Friedman test). The average pH values were 8.3 in surface zone A, 9.0 in intermediate zone B, and 8.9 in deep zone C (Figure 3). High EC values were detected in zone A, and then a constant decrease was observed along with the soil profile. Significant differences in EC were found in the three-zone intervals (*p* < 0.05, Friedman test). The average EC value in surface zone A was 107 mS cm^–1^, 22.1 mS cm^–1^ in intermediate zone B and 14.3 mS cm^–1^ in deep zone C (Figure 3).

### Distribution of C, N, P, and S in the Soil Profile

**FIGURE 4:**
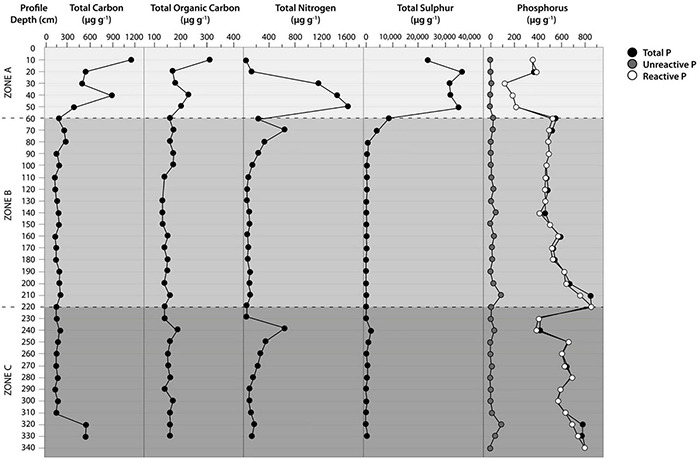
Distribution of the soil profile parameters at the Yungay site in the hyperarid core of the Atacama Desert. Total Carbon (TC), Total Organic Carbon (TOC), Total Nitrogen (TN), Total Sulfur (TS), Total Phosphorus (TP), Reactive Phosphorus (RP), Unreactive Phosphorus (UNP).

The contents of TC in surface zone A varied between 190 and 1125 μg C g^–1^, while in intermediate zone B, the values ranged from 135 to 280 μg C g^–1^. In deep zone C, the values ranged from 140 to 540 μg C g^–1,^ reaching the maximum value in this zone at an interval of 310–330 cm (Figure 4). There were significant differences between the means of TC in the three proposal zones (*p* < 0.05, Friedman test). Concerning the TOC, the values were low along the soil profile (Figure 4). In zone A, TOC ranged between 160 and 310 μg TOC g^–1^ with a high value at 10 cm depth. In zone B, the TOC values were relatively constant, at approximately 140–170 μg TOC g^–1^. Instead, we detected elevated TOC concentrations in deep zone C at 240 and 300 cm depths (190 μg TOC g^–1^). However, there were no significant differences between the means of TOC in the three proposal zones (*p* > 0.05, Friedman test). On the other hand, the TN concentrations generally decreased with increasing soil depth (Figure 4). Thus, TN concentrations were highest in surface zone A (with a maximum value of 1, 594 μg N g^–1^ at 50 cm depth), while in zones B and C, TN decreased from 628 to 39 μg N g^–1^ and 628 to 134 μg N g^–1^, respectively. There were no significant differences between TN means in the three-zone intervals (*p* > 0.05, Friedman test). Similarly, high TS contents were measured in the first 50 cm of soil depth (surface zone A), ranging from 23.9 to 35.3 mg S g^–1^ soil (Figure 4). These values rapidly declined in zones B and C. In intermediate zone B, TS decreased from 4595 to 234 μg Sg^–1^. In deep zone C, TS tended to decrease except for a spike between 230 and 250 cm depth (deep zone C). There were significant differences between TS means in the three-zone intervals (*p* < 0.05, Friedman test).

In contrast to TC, TN, and TS, TP increased in the soil profile. TP showed minimal values in surface zone A (122–216 μg Pg^–1^ at 30–50 cm depth) (Figure 4). Overall, TP concentrations increased into intermediate zone B (from 509 to 840 μg Pg^–1^). In deep zone C, PT decreased at 230-240 cm depth to increase to 783 μg Pg^–1^ at 340 cm depth. There were significant differences between TP means in the three-zone intervals (*p* < 0.05, Friedman test).

Reactive P (or inorganic P) dominated the soil profile (Figure 4), representing over 88% of TP in all samples. The difference between TP and reactive P is explained by the presence of unreactive P (URP) compounds, including condensed phosphates and organic P. Considering the generally low TOC contents, low amounts of organic P could be expected. However, it was possible to detect three small peaks of the URP: 42.7, 87.7 and 87.7 μg URP kg^–1^ at 140, 210, and 320 cm soil depths along with the soil profile. The latter two signals coincided with increasing TOC concentrations in depth. The easily extractable P (the sum of Pi-H_2_O and Pi-NaHCO_3_) only accounted for 1.0–5.6% of the total P (Supplementary Table 2). The highest P was present in the Pi-HCl fraction (> 94%), highlighting that a high P was bound to calcium. Additionally, XANES P speciation confirmed that most P (90-100% of total P) was present as apatite-P (Supplementary Table 3). Some P (< 10% of total P) was Ca-bound organic P; higher concentrations of Pi-H_2_O were observed in intermediate zone B and deep zone C (8.1–20.6 μg Pg^–1^) than in surface zone A (1.9–2.2 μg Pg^–1^). A similar trend was observed for the Pi-NaOH fraction, with high values found in zones B and C (0.8–3.7 μg Pg^–1^) compared with surface zone A (0.3–1.1 μg Pg^–1^).

### Microbial Community Analysis

**FIGURE 5:**
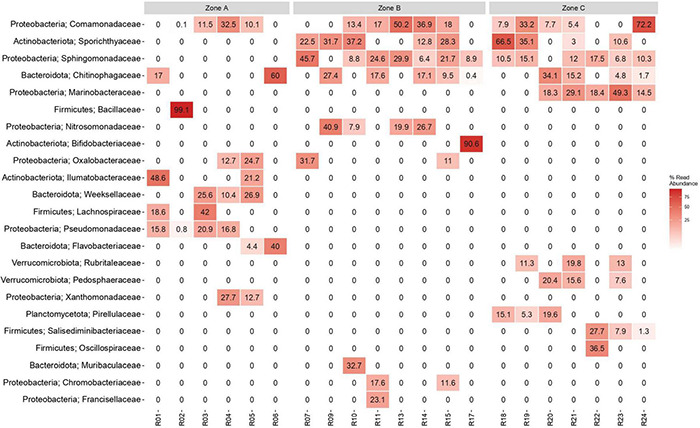
Most abundant bacterial families in samples of the soil profile in the hyperarid core of the Atacama Desert. The heatmap shows the top 10 most abundant families per zone (total 23 families). Taxa are sorted according to overall abundance (%). R01: (0–10 cm); R02: (10–20 cm); R03: (20–30 cm); R04: (30–40 cm); R05: (40–50 cm); R06: (50–60 cm); R07: (60–70 cm); R09: (80–90 cm); R10: (90–100 cm); R11: (100–110 cm); R13: (120–130 cm); R14: (130–140 cm); R15: (140–150 cm); R17: (210–220 cm); R18: (240–250 cm); R19: (250–260 cm); R20: (260–270 cm); R21: (270–280 cm); R22: (290–300 cm); R23: (320–330 cm); R24: (330–340 cm).

The Yungay depth profile showed low DNA concentrations in the range of 1.15–11.8 ng g^–1^ soil. Amplicon sequencing revealed that the phyla *Proteobacteria*, *Actinobacteria*, *Bacteroidota* and *Firmicutes* had the highest prevalence in the soil profile (Figure 5 and Supplementary Figure 2). While *Proteobacteria* and *Bacteroidota* were abundant in all three zones, microbial composition at the family level (Figure 5) and a bipartite network analysis (Supplementary Figure 3) showed that several families and phyla were predominantly detected within one of the three zones, suggesting that communities are structured throughout the soil profile. Soil surface microbial communities were dominated by *Actinobacteria*, *Proteobacteria* and *Firmicutes* (10–20 cm). In general, *Firmicutes* exhibited a slightly greater detection frequency in surface zone A than in zone C, while it was not detected in zone B. Within *Firmicutes*, the occurrence of the families *Lachnospiraceae* and *Bacillaceae* was limited to zone A, while *Oscillospiraceae* and *Salisediminibacter* incerta sedis occurred only in zone C (wetter conditions). Similarly, the actinobacterial families had different niches, with *Sporichthyaceae* being only detected in layers of zones B and C, while *Illumatobacteraceae* were only present in different layers within zone A. *Verrucomicrobiota* and *Planctomycetota* were exclusively present in deep zone C (Figure 5 and Supplementary Figure 3). Moreover, the deepest part of the profile was dominated by *Proteobacteria*, especially by the families *Comamonadaceae* (330–340 cm) and *Marinobacteraceae* (320–330 cm). The family *Sphingomonadaceae* of *Proteobacteria* showed a high abundance in several layers of zone B but a lower abundance in zone C (Figure 5). Some further lower-abundant phyla that were unique for a specific zone were *Deinococcota*, *Halobacterota*, and *Latescibacterota* in surface zone A; *Crenarchaeota*, *Fusobacteriota*, and Sva0485 (deltaproteobacteria) in intermediate zone B; and *Campilobacterota* and *Fervidibacteria* in deep zone C (Supplementary Figure 3). Remarkably, *Cyanobacteria* of the class *Oxyphotobacteria* were detected with low prevalence in various samples across the soil profile (Supplementary Figure 2).

**FIGURE 6:**
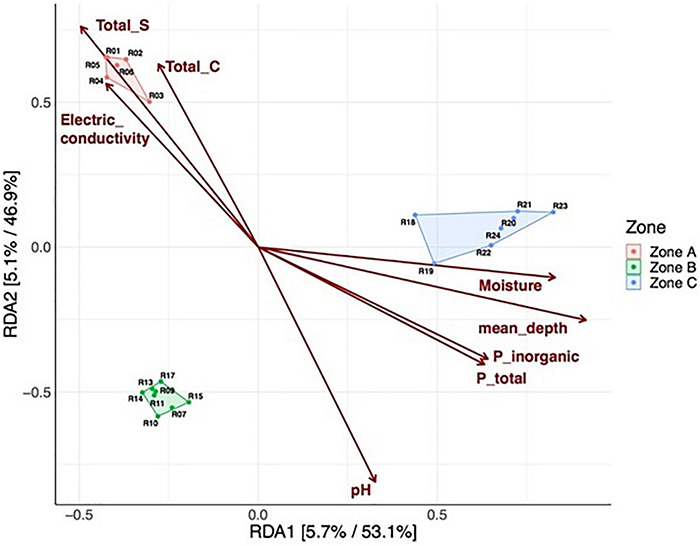
Redundancy analysis (RDA) of Hellinger transformed amplicon sequence variant (ASV) relative abundances. Each point corresponds to a soil sample from a specific depth layer, and its relative distance indicates the level of similarity to all other samples. Polygons and colors label each of the three soil zones. The arrows indicate the explanatory power of the soil parameters concerning the observed variation in community composition. Insignificant soil parameters are not shown. For both axes, the percentages indicate the variance explained in the unconstrained and constrained analysis. Total sulfur (TS), total carbon (TC), total phosphorus (P_Total), unreactive phosphorus or inorganic phosphorus (P_inorganic).

**FIGURE 7:**
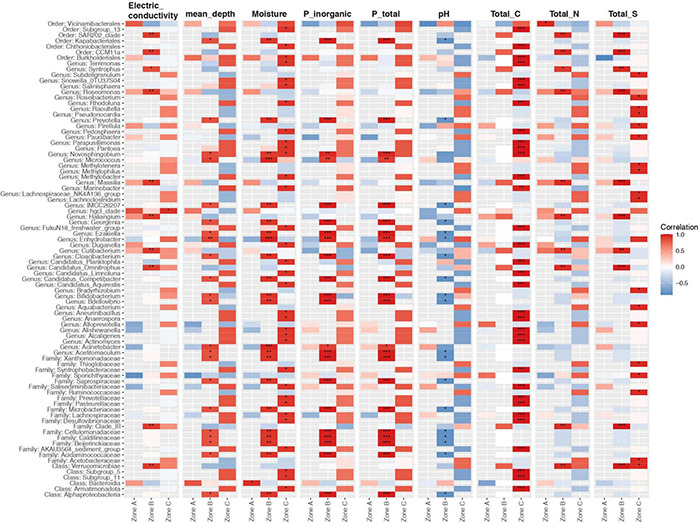
Relationship between the 50 most correlated taxa and soil variables grouped by soil zone. Asterisks show the level of significance (*p value < 0.05, **p value < 0.01, ***p value < 0.001; Pearson correlation). Comparisons were adjusted for multiple testing using the Benjamini-Hochberg correction. Red, blue, and white indicate positive, negative, and no correlation, respectively. Taxa represent the best-hit taxonomic classification.

We also tested to what extent the properties of the soil explained the microbial community structure. We found that microbial communities were to some extent structured by depth, as samples from either zone A, B, or C were more similar within each zone than between zones (Figure 6; polygons). Higher levels of TC, TS, and EC were preferentially associated with microbial communities from zone A, while higher moisture contents, depth, and total and inorganic P were associated with microbial communities from zone C (Figure 6). Finally, we explored associations between the relative abundance of microbial community members and soil variables as a soil zone function (Figure 7). We found statistically significant positive correlations between taxa and soil properties, most of which involved moisture and TC contents (Figure 7). Some taxa were weakly negatively associated with pH value or positively associated with EC as well as TN and TS and PT contents. We also correlated alpha diversity measures to soil properties and observed that the moisture content, TC, pH, and EC correlated positively with microbial richness and diversity (Supplementary Figure 4 and Supplementary Table 4; positive slope; Chao1), while TS and TP contents correlated negatively with diversity (Supplementary Figure 4; negative slope).

## Discussion

### Properties of the Soil Profile and Zonification Proposal

Climatic factors (mainly low precipitation and high temperature) limit biological productivity and activity, chemical reactions, and weathering (Plaza et al., 2018). Consequently, in hyperarid soils, a relatively poor carbon content, low microbial activity, and low soil weathering would be expected (Ewing et al., 2006; Mörchen et al., 2019; Knief et al., 2020). However, in this study, we show that it is possible to find an abundance of MC in deep hyperarid soils, affecting soil and microbial parameters. Then, we proposed three zones (surface zone A, intermediate zone B and deep zone C) considering the significant difference in the MC (*p* < 0.05 Friedman test). These zones also showed significant variations in pH, EC, TC, TS and TP (*p* < 0.05 Friedman test). Instead, at least two zones were similar in terms of TOC and TN (*P* > 0.05 Friedman test). The increase in MC in the depth soil was observed in the soil profile (Figure 3). Recently, wet layers below the surface of the hyperarid core of the Atacama have been reported (Azua-Bustos et al., 2020). Although the origin of water is outside the scope of this study, plausible water deep sources could be related to the unusual rain events that took place during March 2015, as have been indicated by Azua-Bustos et al. (2020). Precipitation events could lead to a reload of deep groundwater system present in the Aguas Blancas basin (Dirección General de Aguas, 2017). It has a low recharge rate, limited to very sporadic rare wet events (Herrera and Custodio, 2014). Whether, larger rainfall events have a longer lasting impact on the moisture content in the deepest part of soils or sediments in the region is still unresolved.

The entire soil profile showed alkaline pH values and high EC, which is frequent in other soils of the Atacama Desert (Connon et al., 2007; Fuentes et al., 2014; Knief et al., 2020). However, slightly lower pH values were observed in surface zone A, while the highest EC was detected in this zone. As expected in these environments, the soil is rich in salt minerals, which result in elevated EC in the entire profile (>5 mS cm^–1^); however, in surface zone A, the soil is exceptionally saline, reaching values over 100 mS cm^–1^. The highest EC value of 185 mS cm^–1^ was recorded at 40 cm depth and was related to salt minerals such as halite, albite, calcite, anorthite, and darapskite (Supplementary Table 1). Decreasing precipitation and increasing evapotranspiration reduce the loss of salts by leaching, leading to the accumulation of calcium carbonate and gypsum and relatively high pH typical of dryland soils (Plaza et al., 2018). The minerals found in the soil profile (Supplementary Table 1) resemble those that have previously been detected in surface and subsurface soils of the Yungay area (Ewing et al., 2006; Fuentes et al., 2014; Azua-Bustos et al., 2020). Complementarily, a soil profile study in the Yungay region by Ewing et al. (2006) revealed a similar stratification of soil with quartz, gypsum, anhydrite, chlorite (0–12 cm), anorthite (39–71 cm) and halite (102–122 cm). However, deeper soil horizons have not been analyzed in a hyperarid desert to date. On the other hand, Crits-Christoph et al. (2013) also found a significant and direct inverse relationship between pH and EC values in surface soils of the hyperarid core in the Atacama Desert. The increased solubility of Ca^2+^ ions would give a possible reason for the negative relationship between pH and EC in saline conditions under ambient atmospheric CO_2_ concentrations that lead to a release of hydrogen ions. Other soil parameters, such as CaCO_3_, gypsum and clay contents, did not appear to have a prominent effect on soil pH as the concentration of soluble Ca^2+^ ions increased (Al-Busaidi and Cookson, 2003).

Interestingly, Azua-Bustos et al. (2020) confirmed the existence of a widespread and sustained phenomenon of subsurface water at Yungay due to the interstratified wet clay-bearing layer. XRD showed that montmorillonite was detected in separate soil intervals in zone B (Supplementary Table 1). Meanwhile, muscovite and chlorite were predominant in deep zone C. These clays retain moisture at greater soil depths and reinforce the findings of Azua-Bustos et al. (2020), who investigated an interstratified wet clay-bearing layer in the upper 30 cm of a soil profile. It contained 42.8 wt% clay, mainly illite–smectite, a group of expandible clays 2:1, such as montmorillonite. Azua-Bustos et al. (2020) stated that these wet clay-rich layers play a significant role in life while preserving biosignatures in the hyperarid core of the Atacama Desert. Furthermore, in these moist smectite-rich layers were even able to detect metabolically active microorganisms protected from extremely harsh conditions at the surface.

In our study, TC was higher than TOC, which was low in the soil profile. Dryland soils store vast amounts of inorganic C. In particular, the soil inorganic C content at any depth up to 2 m is positively related to aridity (Plaza et al., 2018). Valdivia-Silva et al. (2012) indicated that the top 1 m soil layer of hyperarid lands contains ∼11.6 Tg of organic carbon and 344.6 Tg of carbonate carbon. Previous studies in the same place have indicated that the total stored carbon was 30.8-fold that of organic carbon alone (Valdivia-Silva et al., 2012). In zones B and C, it was possible to detect some peaks of TOC. In this sense, Mörchen et al. (2019) demonstrated that C accrual shifted from preferential C enrichment in topsoil to subsoil, thereby providing the potential for deep(er) biosphere food webs and revealing the future need to dig into the soil to discover traces of life in comparable environments. On the other hand, clay minerals in soil have an active role in the OC stock or C sequestration in soils (Zhong et al., 2018; Churchman et al., 2020). Organomineral interactions depend on cation bridges involving Ca ions in neutral to alkaline soils. Various organomineral interactions lead to aggregations of clay particles and organic materials, stabilizing both the soil structure and the carbon compounds within the aggregates (Oades, 1988).

The highest TN concentrations in surface zone A originated from N minerals such as darapskite (37.6%) and nitratine (2.8%) (Figure 4 and Supplementary Table 1). It has been shown that the nitrate deposits present in the Atacama Desert are of atmospheric origin and formed through photochemical reactions (Ericksen, 1981; Melchiorre et al., 2018). Additionally, high contents of TS were measured in the first 50 cm depth (surface zone A); these values rapidly declined to below 500 μg Sg^–1^ in zone B and then stayed below that value throughout the profile, except for a slight spike in TS contents between 230–250 cm depth (zone C). Anhydrous sulfate phases were typical in the surface layers, and the uppermost units had the least water-soluble phases. In the 0–10 cm surface layer, sulfur minerals such as Bassanita (11.6%), anhydrite (4.4%) and gypsum (1.3%) were predominant. Similar results for TOC and S contents were reported for the 0-80 cm soil depth by Warren-Rhodes et al. (2019).

Studies of P dynamics in hyperarid soils have received much less attention, even though P is an essential nutrient for life. Inorganic P (mainly in the Pi-HCl fraction) was dominant around the soil profile, which is in agreement with Wang et al. (2021). However, bioavailable P and colloidal P have been found in the surface sediments of the Atacama Desert, partly even correlating with soil microbial biomass (Warren-Rhodes et al., 2019; Knief et al., 2020; Moradi et al., 2020). Ca-P bonding forms identified by XANES were dominant, as reported earlier by Moradi et al. (2020) using colloid analyses. Higher concentrations of Pi-H_2_O (representing inorganic labile P) were observed in intermediate zone B and deep zone C (8.1–20.6 μg Pg^–1^) (Supplementary Table 2), reflecting that an increase in soil MC increases simultaneously with the availability of P. In this regard, mineralogy, associated subsoil moisture and resulting improvements in available P concentrations founded conditions that enabled the existence of a microbial biosphere within the hyperarid core of the Atacama Desert. Dryland soils also exhibit larger labile inorganic and apatite P contents but are more deficient in organic, occluded, and secondary mineral P (Plaza et al., 2018).

Increased aridity and temperature may also decouple the spatial variability of soil nutrient stocks and cycling in global drylands (Plaza et al., 2018). In the hyperarid core, the surface is devoid of vegetation; it is expected that the cycles of the nutrients carbon (C), nitrogen (N) and phosphorus (P) in the soil are mainly controlled by geochemical factors. This is in contrast with the soil-plant systems in less arid parts of the world. These cycles are coupled with biological (photosynthesis, respiration, and decomposition) and geochemical (physical and chemical weathering) processes. Aridity tends to decrease soil organic C and total N contents to different extents while increasing total and labile inorganic P, which could be related to increased P release by rock physical weathering and minimal P uptake by plants (Plaza et al., 2018).

### Microbial Community Composition

The microbial composition in the soil core was dominated by *Proteobacteria, Actinobacteria, Bacteroidetes* and *Firmicutes* in all studied depth zones (Figure 5 and Supplementary Figures 2, 3). However, community composition varied with soil depth, which is consistent with other studies focusing on bacterial community variations in the surface and subsurface of this harsh environment (Crits-Christoph et al., 2013; Warren-Rhodes et al., 2019). The distribution of phyla in zone A of our study is partially in agreement with previous surface sediment-based studies (Crits-Christoph et al., 2013; Schulze-Makuch et al., 2018; Warren-Rhodes et al., 2019; Knief et al., 2020). Other studies by Neilson et al. (2012) demonstrated that *Actinobacteria* and *Chloroflexi* dominate soil microbial communities in the hyperarid margin of the Atacama Desert, with extremely low levels of *Acidobacteria, Alpha-* and *Betaproteobacteria*. Neilson et al. (2017) also found that bacterial communities in soils of the Atacama Desert are mostly dominated by *Actinobacteria*, *Chloroflexi, Proteobacteria* and *Gemmatimonadetes*. Recently, Knief et al. (2020) showed the dominance of different groups of *Actinobacteria*, *Proteobacteria* and *Chloroflexi* in soils of the Atacama Desert. Connon et al. (2007) detected *Actinobacteria, Proteobacteria* and *Firmicutes* in Yungay surface soils (0–2 cm and 2–20 cm). Thus, this microbial description is in accordance with the results of our study. Recently, bacterial, and archaeal communities from hypolithic microhabitats were analyzed in the Atacama Desert to specifically identify the potentially viable microbiota (intracellular DNA versus extracellular DNA), showing habitat-specific communities dominated by bacteria. Proteobacteria were almost exclusively identified in the extracellular DNA pool in Yungay Salar halite nodules and gypsum crusts (representing an indicator for a previously existing community in this location), and *Cyanobacteria* showed the most abundant intracellular DNA in these hypolithic environments (Schulze-Makuch et al., 2021). In our case, the presence of active microbiota in deep soil remains to be addressed in future studies because we must determine if the extraction method used affected the intra- and extracellular DNA amount. The microbiological findings mentioned above show that although endolithic and hypolithic communities have been extensively studied in hyperarid surface soils of the Atacama Desert near the Yungay area (e.g., Warren-Rhodes et al., 2006; Schulze-Makuch et al., 2021), our results indicate that *Cyanobacteria* of the class *Oxyphotobacteri*a were detected with low prevalence in some of the samples of the soil profile (Supplementary Figure 2). Despite ecological range of *Cyanobacteria* appeared to be restricted to environments with at least occasional expose to sunlight, their presence also extends down to the deep terrestrial biosphere (Puente-Sánchez et al., 2018). A few studies have reported the presence of *Cyanobacteria* in deep subsurface environments (e.g., Kormas et al., 2003; Rastogi et al., 2010; Hubalek et al., 2016; Puente-Sánchez et al., 2018). The discussion of its origin is limited, in which it has been proposed that bloom of aquatic *Cyanobacteria* had been trapped thousands of years ago into a groundwater aquifer with no further connection with the surface (Hubalek et al., 2016), and that the presence of *Cyanobacteria* in the continental subsurface were related to surface rock-dwelling lineages known for their high tolerance to environmental and nutritional stress (Puente-Sánchez et al., 2018). In our case, it is difficult to indicate the presence of living *Cyanobacteria* in the soil samples but this type of microorganisms in the depth soil could be related to microbial transport due surface run off rainfall events indicated by Azua-Bustos et al. (2020), and we also suggest the discovery of ancient extracellular DNA.

Moreover, our results suggest that higher moisture content, TC, pH, and EC contents favored microbial communities with more taxa that were more evenly represented (Supplementary Figure 4 and Supplementary Table 4). Soil moisture is essential for bacterial diversity in desert ecosystems (Bottos et al., 2020). Additionally, moisture could be ancillary to other factors in shaping bacterial diversity. Moreover, the clay-rich layers can act as a possible “water reservoir” and help shape microbial life conditions and associated “hotspot” biosignals at greater soil depths.

The salinity of the soil, reflected by the electrical conductivity, was high along the entire soil profile (Figure 3). It seems to shape bacterial communities due to intense selective pressure, as few bacteria are capable of growing over large gradients of salt concentrations. We measured exceptionally high EC values (over 25 to 187 mS cm^–1^) in surface zone A, while in intermediary zone B and depth zone C, the EC values fluctuated between 5 and 24 mS cm^–1^. However, we did not observe a particular accumulation of halophilic taxa in zone A; rather, they were present in all zones. Nevertheless, EC had a significant effect on the bacterial community structure (Figure 6 and Supplementary Figure 4). Interestingly, some of the families present in the soil profile are known to include halophilic taxa or have been shown to be present in other saline environments, such as *Proteobacteria* (*Comamonadaceae*, *Marinobacteraceae*, *Nitrosomonadaceae* and *Pseudomonadaceae*), *Actinobacteria* (*Bifidobactericeae* and *Sporichthyaceae*), and *Firmicutes* (*Bacilliaceae* and *Salisediminibacteriaceae*), which have been detected in different saline environments (e.g., Jiang et al., 2012; Crognale et al., 2013; Mirete et al., 2015; Newton et al., 2018; Remonsellez et al., 2018; Zhang et al., 2020). Moreover, members of the families *Chitinophagaceae*, *Ilumatobacteraceae*, *Rubritaleaceae*, *Pedosphaeraceae*, and *Pirellulaceae* have been detected in various soils and sediments, such as desert surfaces (China), arid biological crusts (United States), and hypersaline lagoons (Chile) (Moquin et al., 2012; An et al., 2013; Fernandez et al., 2016; Asem et al., 2018; Li et al., 2021). Interestingly, the cultivation of clay-rich soil from subsurface samples of Yungay highlighted novel halotolerant bacteria related to *Oceanobacillus*, *Lysinibacillus*, *Virgibacillus*, *Halobacillus* and *Bacillus* (Azua-Bustos et al., 2020).

On the other hand, microbial communities were related to TP and IP (Figure 6). In this case, the alkaline pH values, P bonds to Ca as apatite-P (by XANES analyses), and the predominance of the Pi-HCl fraction indicates low P availability in the soil profile. However, in intermediate zone B and deep zone C, slight major P availability was revelated by high values of soluble Pi, as shown in fraction Pi-H_2_O (Supplementary Table 2). Studies by Oliverio et al. (2020) suggest that the scarce availability of soil P likely constrains microbial growth, favoring slower growing oligotrophic microorganisms that can survive under nutrient limitations, suggesting that bacteria in low P soils may have strategies to permit efficient phosphate uptake, including the utilization of organic P compounds.

As in our study, several authors have found an association between microbial richness and diversity in the Atacama Desert’s hyperarid surface soil with water availability and relative humidity (Crits-Christoph et al., 2013; Neilson et al., 2017; Schulze-Makuch et al., 2018; Knief et al., 2020). Moreover, Warren-Rhodes et al. (2019) highlighted the effect of water availability and geochemical parameters down to 80 cm depth. This study shows for the first time that this phenomenon also occurs at soil depths up to 340 cm. To date, the work of Schulze-Makuch et al. (2018) and Warren-Rhodes et al. (2019) have proposed a correlation between microbial community patterns and soil parameters and depths. In this context, our results suggest that soil properties such as moisture content and TC content strongly influence species richness, diversity, community composition, and specific members of microbial communities but that these relationships do not end in the very surface soil but extend several meters deep. Therefore, the search for life in extreme environments should consider soil as a three-dimensional ecosystem. This would increase the prospects of finding moisture hotspots at such extended soil depths, even if hyperarid conditions dominate the land surface.

## Conclusion

The differential but ‘segmented’ physico-chemical and mineralogical features observed in our Yungay profile evidenced that it was possible at greater soil depths to find favorable and under-explored hotspots for microbial life in hyperarid Atacama soils. We found that the combined effects of depth, moisture, EC, pH, TC and P availability played a critical role in driving the composition and/or diversity of microbial communities in our hyperarid desert profile. Clearly, extensive and diverse microbial life remains to be discovered at greater soil depths within the hyperarid Atacama Desert, and by inference other extreme environments (including extraterrestrial planets).

## Data Availability Statement

The datasets presented in this study can be found in online repositories. The names of the repository/repositories and accession number(s) can be found below: https://www.ncbi.nlm.nih.gov/genbank/, PRJNA638921.

## Author Contributions

BF, FR, and RB conceived and designed the study. BF, AC, FG, FA, FR, GM, DC, and RB performed the field work. AC, FG, DC, GM, JP, and WK performed the experimental procedures and laboratory work. JA, EC-N, CK, and FR analyzed sequencing data. BF, EC-N, FR, and RB wrote the manuscript. RM, WA, CK, EK, and CS reviewed and edited the manuscript. All authors read and approved the final manuscript.

## Conflict of Interest

The authors declare that the research was conducted in the absence of any commercial or financial relationships that could be construed as a potential conflict of interest.

## Publisher’s Note

All claims expressed in this article are solely those of the authors and do not necessarily represent those of their affiliated organizations, or those of the publisher, the editors and the reviewers. Any product that may be evaluated in this article, or claim that may be made by its manufacturer, is not guaranteed or endorsed by the publisher.

## Footnotes

1
https://github.com/umerijaz/microbiomeSeq
