# Supplementary material for: Influence of Physical-Chemical Soil Parameters on Microbiota Composition and Diversity in a Deep Hyperarid Core of the Atacama Desert
Source: Front Microbiol. 2022 Feb 7;12:794743. doi: 10.3389/fmicb.2021.794743 (PMC8859261; doi:10.3389/fmicb.2021.794743)
Supplement: Supplementary file 1 [file Table_1.DOCX]

**SUPPLEMENTARY MATERIAL**

**Supplementary Figure 1.** Geological description of the soil profile in the hyper-arid core of the Atacama Desert.


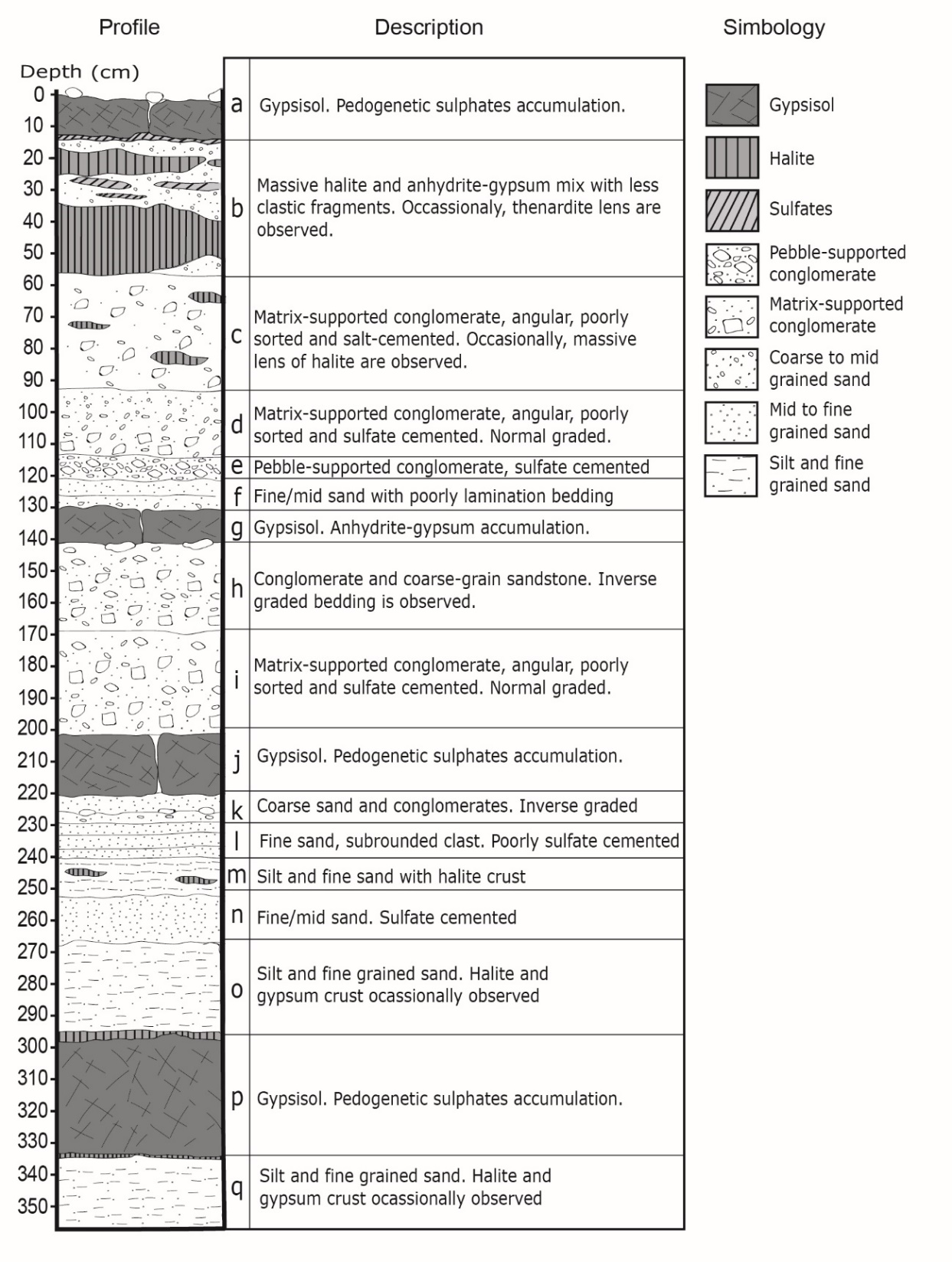


**Supplementary Figure 2.** Scatterplots for phylum prevalence as a function of total read abundance. Each point represents an ASV belonging to a particular phylum and the dotted line marks an arbitrary prevalence threshold of 5% of the samples. Phyla with less than 5 members are not presented.


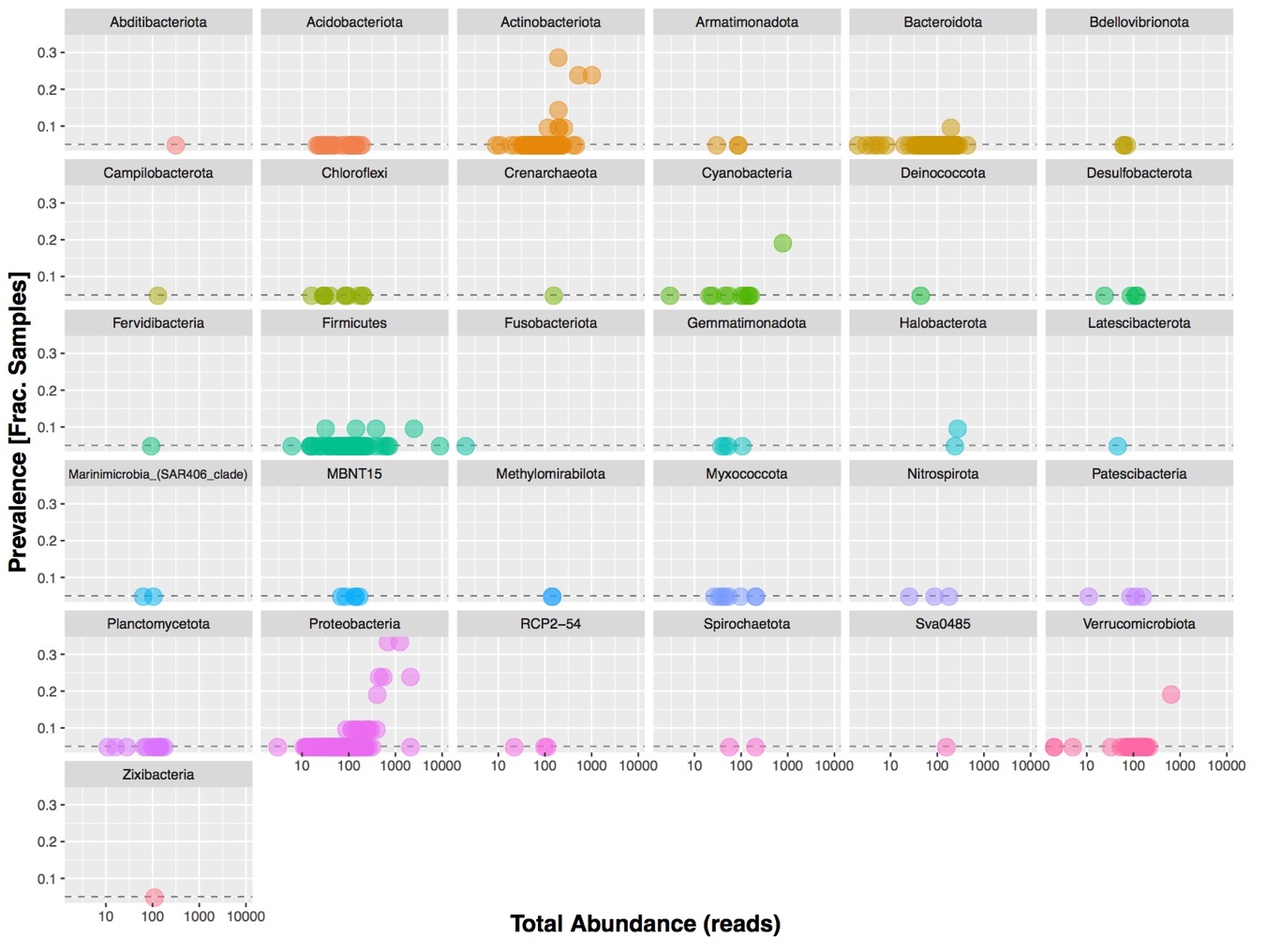


**Supplementary Figure 3.** Bipartite network shows associations among soil profile zone and occurrence of bacterial phyla. Zone boxes and lines are colored to indicate associations with a phylum. Box width is proportional to the number of sequences corresponding to each phylum (bottom) or the proportion of reads obtained from each soil zone (Top). Names of phyla given in columns correspond to the sections in the horizontal black bar from left to right.

**Supplementary Figure 4.** Scatter plots of relationships between alpha diversity indices as a function of soil variables. Tick marks above the X axis represent samples and the blue line is the adjusted generalized linear model (GLM) with bands representing 95% confidence intervals. *P* values per each effect are indicated within the plots. Alpha diversity is assessed as estimated species richness (Chao1) and diversity (Shannon). We also assessed diversity under low sample completeness using Fisher’s alpha (not shown), which resulted in similar results as Chao1.


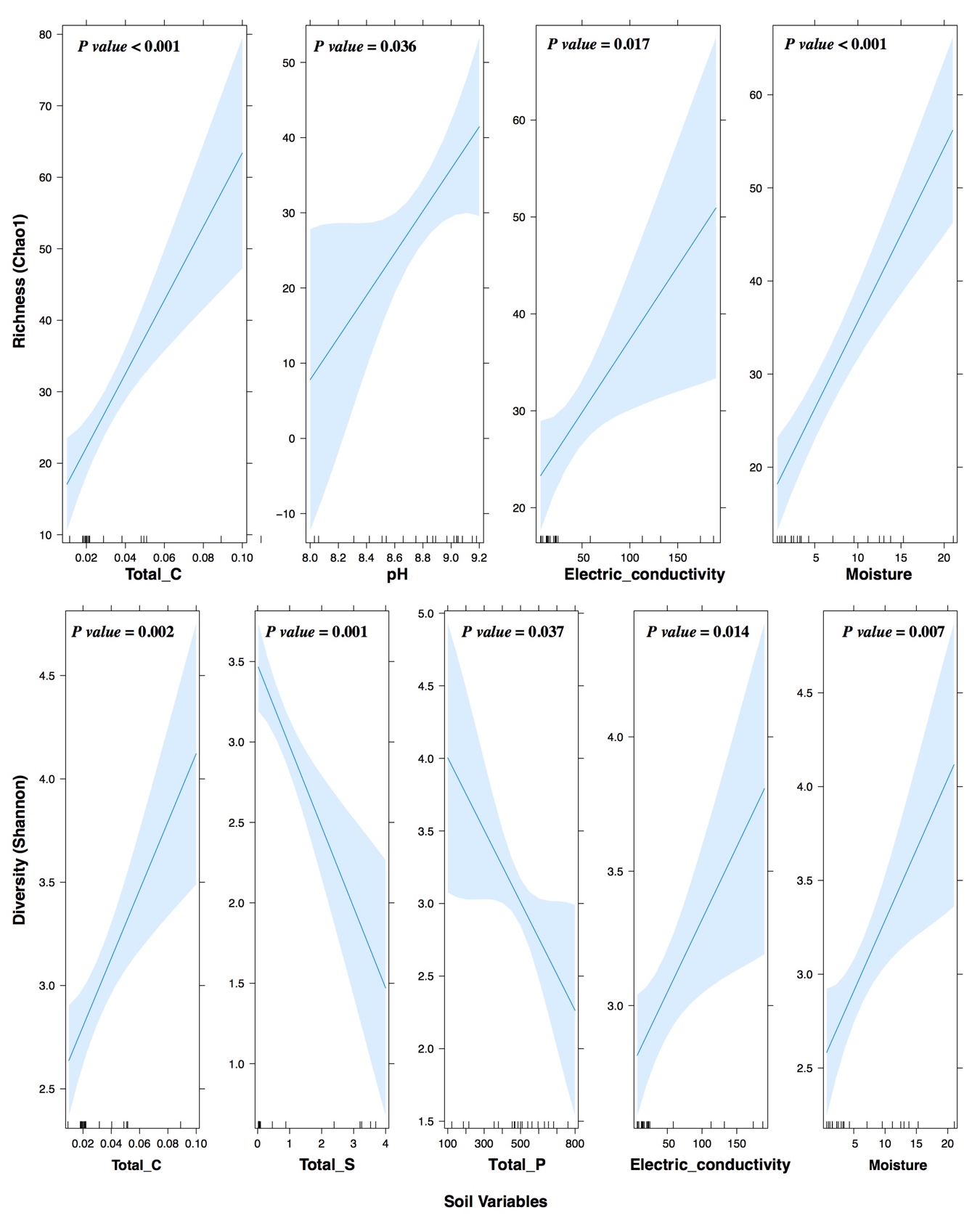


**Supplementary Table 1**. X-ray diffraction analysis (XRD) of the soil profile at the study site in Yungay

| Compound (~%) | Chemical composition | Depth (cm) | | | | | | | | | | |
| --- | --- | --- | --- | --- | --- | --- | --- | --- | --- | --- | --- | --- |
|  |  | Zone A | | Zone B | | | | | | Zone C | | |
|  |  | 0 - 10 | 40 - 50 | 60 - 70 | 80-90 | 100-110 | 140-150 | 170-180 | 200-210 | 230-240 | 270-280 | 330-340 |
| Quartz | SiO_2_ | 30.6 | 5.5 | 25.0 | 29.8 | 26.5 | 38.9 | 31.8 | 25.4 | 34.2 | 14.8 | 10.8 |
| Halita | NaCl | 1.7 | 46.0 | 2.8 | 3.0 | 0.9 | 1.2 | 3.8 | 4.5 | 1.9 | 5.4 | 3.5 |
| Albite | $\text{NaAl}\text{Si}_{\text{3}}\text{O}_{\text{8}}$ | - | 9.7 | - | - | - | - | - | - | - | - | - |
| Cacite albite | $\left( \text{Na,Ca} \right)\text{Al}\text{(Si,Al)}_{\text{3}}\text{O}_{\text{8}}$ | 25.0 | - | - | 18.5 | 15.0 | 2.3 | 26.1 | 19.7 | 31.1 | 26.2 | 17.2 |
| Anorthoclase | $(\text{Na,K)}{\left( \text{Si}_{\text{3}}\text{Al} \right)\text{O}}_{\text{8}}$ | 7.8 | - | - | - | - | - | 3.0 | - | 3.2 | - | 8.8 |
| Anorthite | $(\text{Ca,Na)}\text{(Si,Al)}_{\text{4}}\text{O}_{\text{8}}$ | - | - | 39.8 | 28.8 | 34.4 | 29.7 | - | - | - | - | - |
| Anhydrite | $\text{CaS}\text{O}_{\text{4}}$ | 4.4 | - | - | - | - | - | - | - | - | - | - |
| Bassanite | $\text{CaS}\text{O}_{\text{4}}\cdot0,5\text{H}_{\text{2}}\text{O}$ | 11.6 | - | - | - | - | - | - | - | - | - | - |
| Hydrochlorborite | $\text{Ca}_{\text{2}}\text{B}_{\text{4}}\text{O}_{\text{4}}\text{(OH)}_{\text{7}}\text{Cl}_{\text{7}}\text{∙}\text{H}_{\text{2}}\text{O}$ | 1.1 | - | 1.9 | - | - | - | - | - | 0.4 | - | - |
| Orthoclase | $\text{KAl}\text{Si}_{\text{3}}\text{O}_{\text{8}}$ | 12.7 | - | 17.1 | 17.0 | 16.7 | 24.0 | 26.2 | 23.2 | 25.0 | 20.4 | 18.2 |
| Magnetite | $\text{Fe}_{\text{3}}\text{O}_{\text{4}}$ | 0.7 | - | 1.1 | - | - | - | - | - | - | - | - |
| Hematite | Fe_2_O_3_ | - | - | - | - | 0.7 | 1.2 | - | - | - | - | - |
| Gypsum | $\text{CaS}\text{O}_{\text{4}}\text{·2H}_{\text{2}}\text{O}$ | 1.3 | 0.2 | - | - | - | - | - | - | - | - | - |
| Ilmenita | $\text{FeTi}\text{O}_{\text{3}}$ | 1.1 | - | - | - | - | - | - | - | - | - | - |
| Chlorite (1MIIb) | $\text{(}\text{Mg}_{\text{5}}\text{Al)}\text{(Si,Al)}_{\text{4}}\text{O}_{\text{10}}\text{(OH)}_{\text{8}}$ | 2.2 | - | 2.4 | - | - | - | - | - | 4.1 | 3.7 | 3.8 |
| Darapskite | $\text{Na}_{\text{3}}\text{(N}\text{O}_{\text{3}}\text{)(S}\text{O}_{\text{4}}\text{)∙}\text{H}_{\text{2}}\text{O}$ | - | 37.6 | - | - | - | - | - | - | - | - | - |
| Calcite | $\text{CaC}\text{O}_{\text{3}}$ | - | 1.0 | - | - | - | - | - | - | - | - | - |
| Nitratine | $\text{NaN}\text{O}_{\text{3}}$ | - | - | 2.8 | - | - | - | - | - | - | - | - |
| Loeweita | $\text{Na}_{\text{12}}\text{Mg}_{\text{7}}{\text{(S}\text{O}_{\text{4}}\text{)}}_{\text{13}}\text{∙15}\text{H}_{\text{2}}\text{O}$ | - | - | 5.0 | - | - | - | - | - | - | - | - |
| Montmorillonite (15A) | $\text{Na}_{\text{0,3}}\text{(Al,Mg)}_{\text{2}}\text{Si}_{\text{4}}\text{O}_{\text{10}}\text{(OH)}_{\text{2}}\text{∙4}\text{H}_{\text{2}}\text{O}$ | - | - | 2.2 | - | - | - | 9.1 | - | - | - | - |
| Mordenite | $\text{(}\text{Na}_{\text{2}}\text{,Ca,}\text{K}_{\text{2}}\text{)}\text{Al}_{\text{2}}\text{Si}_{\text{10}}\text{O}_{\text{24}}\text{∙7}\text{H}_{\text{2}}\text{O}$ | - | - | - |  |  |  | - | 4.9 | - | - | - |
| Analcime | $\text{Na}_{\text{0,92}}\text{(}\text{Al}_{\text{0,84}}\text{Si}_{\text{2,16}}\text{O}_{\text{6}}\text{)(}\text{H}_{\text{2}}\text{O})$ | - | - | - | 2.8 | 3.0 | 2.1 | - | 2.3 | - | - | - |
| Bloedite | $\text{Na}_{\text{2}}\text{Mg}\left( \text{S}\text{O}_{\text{4}} \right)_{\text{2}}{\text{(}\text{H}_{\text{2}}\text{O)}}_{\text{4}}$ | - | - | - | - | - | - | - | 20.1 | - | - | - |
| Muscovite IT M RG syn | $\text{K}\text{Al}_{\text{2}}\text{(}\text{Si}_{\text{3}}\text{Al}\text{O}_{\text{10}}\text{)}\text{(OH)}_{\text{2}}$ | - | - | - | - | - | - | - | - | - | 26.0 | - |
| Muscovite-1M, syn | $\text{K}\text{Al}_{\text{2}}\text{(}\text{Si}_{\text{3}}\text{Al}\text{O}_{\text{10}}\text{)}\text{(OH)}_{\text{2}}$ | - | - | - | - | - | - | - | - | - | - | 37.8 |
| Muscovite (2M1) | $\text{K}\text{Al}_{\text{2}}\text{(}\text{Si}_{\text{3}}\text{Al}\text{O}_{\text{10}}\text{)}\text{(OH,F)}_{\text{2}}$ | - | - | - | - | 2.8 | - | - | - | - | - | - |
| Heulandite | $\text{Ca(}\text{Si}_{\text{7}}\text{Al}_{\text{2}}\text{)}\text{O}_{\text{18}}\text{∙6}\text{H}_{\text{2}}\text{O}$ | - | - | - | - | - | - | - | - | - | 3.5 | - |

**Supplementary Table 2.** Phosphorus fractionation (mg kg^-1^) in soil profile of Yungay site. Data shows mean ± standard deviation.

| Zones | Depth  (cm) | Pi-H_2_0  (mg kg^-1^) | Pi-NaHCO_3_  (mg kg^-1^) | Pi-NaOH  (mg kg^-1^) | Pi-HCl  (mg kg^-1^) | P-Res  (mg kg^-1^) | Total P Hedley  (mg kg^-1^) |
| --- | --- | --- | --- | --- | --- | --- | --- |
| Zone A  (0-60 cm) | 0-10 | 1.9 ± 0.0 | 6.2 ± 0.1 | 0.3 ± 0.0 | 309 ± 2 | 51 ± 1 | 368 ± 4 |
|  | 40-50 | 2.2 ± 0.5 | 4.1 ± 1.5 | 0.3 ± 0.0 | 218 ± 11 | 99 ± 18 | 324 ± 9 |
| Zone B  (61-220 cm) | 60-70 | 2.9 ± 0.6 | 2.8 ± 0.6 | 1.1 ± 0.1 | 453 ± 29 | 60 ± 3 | 521 ± 26 |
|  | 170-180 | 20.6 ± 0.7 | 4.4 ± 0.3 | 0.8 ± 0.2 | 466 ± 5 | 41 ± 2 | 532 ± 3 |
|  | 200-210 | 11.0 ± 2.0 | 3.4 ± 0.0 | 3.4 ± 0.2 | 635 ± 5 | 78 ± 2 | 731 ± 4 |
| Zone C  (221-340 cm) | 230-240 | 16. 6 ± 2.5 | 5.5 ± 0.3 | 2.4 ± 0.1 | 315± 6 | 24 ± 1 | 363 ± 8 |
|  | 270-280 | 8.1 ± 0.9 | 1.8 ± 0.3 | 2.4 ± 0.4 | 520 ± 15 | 93 ± 15 | 626 ± 23 |
|  | 330-340 | 16.4 ±1.6 | 3.8 ± 0.1 | 3.7 ± 0.6 | 600 ± 3 | 145 ± 13 | 769 ± 19 |

**Supplementary Table 3.** XANES data from Yungay area in the hyper-arid core in the Atacama Desert

| Depth  (cm) | P_tot_  mg kg^-1^ | XANES  yes/no | Apatite  % P_total_ | Ca-bound organic P  % P_total_ | Other P-forms  % P_total_ |
| --- | --- | --- | --- | --- | --- |
| 0-10 | 330 | No | n.a. | n.a. | n.a. |
| 30-40 | 183 | No | n.a. | n.a. | n.a. |
| 60-70 | 509 | Yes | 93 | 7 | 0* |
| 110-120 | 476 | Yes | 90 | 10 | 0* |
| 130-140 | 454 | Yes | 91 | 9 | 0* |
| 150-160 | 584 | Yes | 92 | 8** | 0* |
| 200-210 | 835 | Yes | 91 | 9 | 0* |
| 230-240 | 410 | Yes | 89 | 10 | 0* |
| 260-270 | 631 | Yes | 100 | 0* | 0* |
| 310-320 | 769 | Yes | 94 | 6 | 0* |
|  |  |  |  |  |  |

n.a. = not applicable

* below quantification limit of P K-edge XANES spectroscopy (5%of total P; Werner & Prietzel, 2016)

** originally indicated as free organic P (IHP).

**Supplementary Table 4.** Results of three Generalized Linear models (GLM), applied to identify significant soil variable predictors on bacterial alpha diversity. In all cases, a Gaussian distribution was used. Only significant findings are reported.

| Predictors | Estimates | Chao1 *CI* | p | Estimates | Shannon *CI* | p | Estimates | Fisher *CI* | p |
| --- | --- | --- | --- | --- | --- | --- | --- | --- | --- |
| (Intercept) | 9.41 | 1.99 – 16.83 | 0.024 | 2.52 | 2.20 – 2.84 | <0.001 | 1.42 | 0.17 – 2.66 | 0.039 |
| Moisture | 1.52 | 0.92 – 2.12 | <0.001 |  |  |  | 0.22 | 0.12 – 0.32 | 0.001 |
| Total_C | 300.59 | 166.60 – 434.59 | <0.001 | 19.25 | 9.31 – 29.19 | 0,002 | 49.77 | 27.33 – 72.21 | <0.001 |
| Total_N |  |  |  | 10.89 | 5.30 – 16.47 | 0,002 |  |  |  |
| Total_S |  |  |  | -0.59 | -0.84 – -0.35 | <0.001 |  |  |  |
| Observations | 20 |  |  | 20 |  |  | 20 |  |  |
| R^2^ Nagelkerke | 1.000 |  |  | 0.642 |  |  | 0.928 |  |  |
